# Preventing falls in older multifocal glasses wearers by providing single-lens distance glasses: the protocol for the VISIBLE randomised controlled trial

**DOI:** 10.1186/1471-2318-9-10

**Published:** 2009-03-26

**Authors:** Mark J Haran, Stephen R Lord, Ian D Cameron, Rebecca Q Ivers, Judy M Simpson, Bonsan B Lee, Mamta Porwal, Marcella MS Kwan, Connie Severino

**Affiliations:** 1 Department of Aged Care and Rehabilitation, Royal North Shore Hospital, St Leonards, Sydney, NSW, Australia; 2 Prince of Wales Medical Research Institute, University of New South Wales, Sydney, NSW, Australia; 3 Rehabilitation Studies Unit, University of Sydney 2006, Sydney, NSW, Australia; 4 Injury Division, The George Institute for International Health, The University of Sydney, Sydney, NSW, Australia; 5 School of Public Health, The University of Sydney, Sydney, NSW, Australia; 6 Department of Rehabilitation, Prince of Wales Hospital, Sydney, NSW, Australia; 7 School of Public Health and Community Medicine, The University of New South Wales, Sydney, NSW, Australia

## Abstract

**Background:**

Recent research has shown that wearing multifocal glasses increases the risk of trips and falls in older people. The aim of this study is to determine whether the provision of single-lens distance glasses to older multifocal glasses wearers, with recommendations for wearing them for walking and outdoor activities, can prevent falls. We will also measure the effect of the intervention on health status, lifestyle activities and fear of falling, as well as the extent of adherence to the program.

**Methods/Design:**

Approximately 580 older people who are regular wearers of multifocal glasses people will be recruited. Participants will be randomly allocated to either an intervention group (provision of single lens glasses, with counselling and advice about appropriate use) or a control group (usual care). The primary outcome measure will be falls (measured with 13 monthly calendars). Secondary measures will be quality of life, falls efficacy, physical activity levels and adverse events.

**Discussions:**

The study will determine the impact of providing single-lens glasses, with advice about appropriate use, on preventing falls in older regular wearers of multifocal glasses. This pragmatic intervention, if found to be effective, will guide practitioners with regard to recommending appropriate glasses for minimising the risk of falls in older people.

**Trial Registration:**

The protocol for this study was registered with the Clinical Trials.gov Protocol Registration System on June 7^th ^2006 (#350855).

## Background

Refractive error due to presbyopia is the most prevalent form of visual impairment in older people [1]. Presbyopia is a visual condition in which the crystalline lens of the eye loses its flexibility, making focusing on close objects difficult [2]. To correct for presbyopia, older people are either prescribed separate single lens glasses for distant and near vision, or for convenience, a single pair of multifocal (bifocal, trifocal or progressive lens) glasses. Multifocal glasses have specific benefits for tasks that require changes in focal length, including everyday tasks of driving, shopping and cooking. However, multifocal glasses also have certain disadvantages as outlined below.

Bifocal glasses have optical defects, such as prismatic jump at the top of the reading segment, which causes an apparent displacement of fixed objects [2,3]. The lower lenses of all types of multifocal glasses blur distant objects in the lower visual field and this factor, in particular, may represent a significant problem for older people [3,4]. Multifocal glasses have been shown to impair distant depth perception and contrast sensitivity [5] and two recent studies have found that multifocal glasses impair step negotiation and the accuracy of foot placement when stepping onto a raised surface in older people [6,7]. These studies reported that when wearing multifocal glasses versus single-lens glasses, older people displayed more variability in minimum vertical toe clearance when stepping up [7] and in foot placement (lead or trail toe-to-platform distance) when negotiating a raised surface [6]. Contacts with the raised platform's edge [7] and trips [6] only occurred in the multifocal glasses conditions.

Other studies have reported significant associations between wearing multifocal glasses and tripping incidents [8,9] and a prospective study of 156 older people found that multifocal glasses wearers were significantly more likely to fall during 12 months of follow-up, after adjusting for age and known physiological risk factors for falls [5]. Findings from this study also indicated that multifocal glasses wearers were more likely to fall when outside their homes, and when walking up or down stairs. The population attributable risks of regular multifocal glasses use were 35% for any falls and 41% for falls outside the home.

To date, there is no evidence that falls can be prevented by restricting the use of multifocal glasses. To address this need we are conducting the Visual Intervention Strategy Incorporating Bifocal & Long-distance Eyewear (VISIBLE) study. The primary aim of this randomized controlled trial is to determine whether the provision of supplementary single-lens distance glasses to elderly multifocal glasses wearers, together with recommendations for wearing them for standing and outdoor activities, can reduce falling rates over a 13-month period. Secondary aims include determining whether, as a result of fewer falls, the intervention has beneficial effects for physical and emotional wellbeing, falls efficacy and activity levels, and whether a range of physical, psychological and socio-demographic factors can predict compliance in the intervention group.

## Methods

### Participants

Participants will be recruited from the following sources: sampling of older people from the electoral roll in northern Sydney, residents of retirement (self-care apartment) villages in the Central Coast, Blue Mountains and Illawarra regions of New South Wales, outpatients and inpatients discharged from rehabilitation and orthopaedic wards at hospitals within northern and eastern Sydney, and responders to newspaper advertisements and media coverage.

Eligibility will be assessed primarily with a telephone screening questionnaire. Participants will be eligible for the trial if they are at a relatively high risk of falls defined as (a) aged 65–79 years with a Timed Up and Go Test (TUG) of 15+ seconds [10] and/or a history of one or more falls in the last 12 months or b) aged 80 years and over. Other inclusion criteria comprise: a user of bifocal, trifocal or progressive-lens glasses 3+ times/week when walking outdoors; having had a review by an optometrist or ophthalmologist in the last 12 months; not currently using single-lens distance glasses; and indicating that they are at least 'quite confident' of complying with the study recommendations.

People will be ineligible if they: reside in a high-care residential facility, have a cognitive impairment (a Mini-Mental State Examination (MMSE) [11] score of less than 24), a severe visual impairment (Melbourne Edge Test score of <16 dB) [12], insufficient English language skills to understand the assessment and/or intervention procedures, have planned ophthalmic surgery in the next 12 months, or have an unstable medical condition at enrolment.

**Figure 1:**
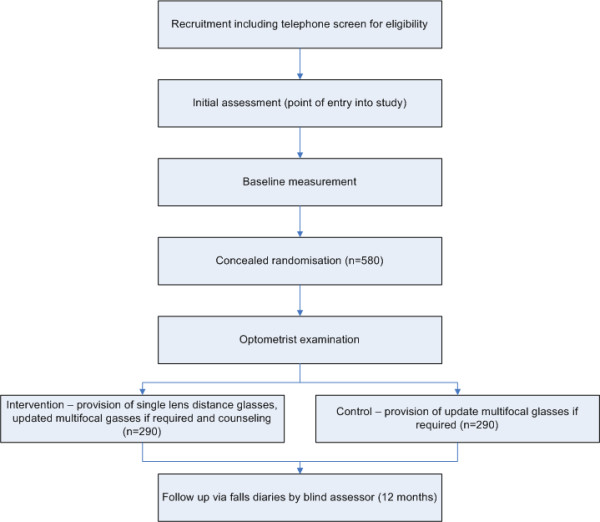
**Flow of participants through the trial**.

The University of New South Wales Human Research Ethics Committee (HREC) approved the study protocol (HREC Number 04229) as have ethics committees at the participating hospitals. The participant flow diagram is presented as figure 1 (Figure 1).

### Initial Assessment

Initially the study aims will be explained, and then the participants will be asked if they feel confident that they could manage and wear two pairs of glasses if randomised to the intervention. Only participants who answer 'very or quite confident', as opposed to 'not confident' or 'very unconfident', will undergo consent, assessments and randomisation. The approach of targeting the intervention to those who consider it to be reasonable and manageable is based on strong evidence from hip protector trials [13].

Demographic details and potential confounders will be recorded, including age, gender, living alone status, MMSE [10], number of falls in past year, number and type of medications, physical activity levels, gait aids, medical conditions relevant to falls (e.g. arthritis, syncope, diabetes and depression), current glasses use and ocular history. Baseline lifestyle activities (using the Adelaide Activities Profile (AAP)) [14], health status (SF-12) [15], and falls efficacy (Falls Efficacy Scale – International (FES-I scale) [16] will be measured.

The following vision and physical assessments will then be carried out. Corrected and uncorrected uniocular and binocular visual acuity will be assessed using a 3-metre logMAR chart [17]. Falls risk will be measured with the short-form Physiological Profile Assessment (PPA) [18] which includes five validated measures of physiological falls risk (visual contrast sensitivity, postural sway, quadriceps strength, reaction time and lower limb proprioception). In multivariate models these variables provide an overall falls risk score, and can predict those at risk of falling with 75% accuracy in community and retirement village settings. Functional mobility will be assessed with the TUG test [10], and the sit-to-stand test [19].

### Randomisation

After completion of the initial assessment, participants will be formally entered into the study and randomised to intervention or control groups. Participants will be stratified by assessment site (Greenwich or Prince of Wales Hospitals) and recruitment source (hospital or community contact).

Then each stratum will be randomly allocated in permuted blocks of 10 using sequentially-numbered opaque envelopes containing group assignment.

### Intervention

Following the initial assessment, participants in the intervention group will have an optometrist examination that will include measurement of objective refraction. These participants will then be prescribed a pair of single-lens distance glasses. For the estimated 80% of intervention participants who will not need an update of multifocal lens prescription (n = 234), the correction for the single-lens glasses will be matched with the prescription of the upper segment of their pre-trial multifocal glasses. For the expected 20% of participants (n = 56) who have had a significant change in distance correction since the most recent prescription [defined as a change of +/- 0.5 dioptres for refractive error (using the spherical equivalent: sphere + half cylinder), or more than 0.75 dioptres for astigmatism (measured in minus cylinders) and at least +0.50 dioptres change in the reading addition], updated multifocal lenses will be provided. The distance component of the updated multifocal correction will then be used for the prescription of new single-lens distance glasses, thereby making the transition between the two types of glasses easier.

**Figure 2:**
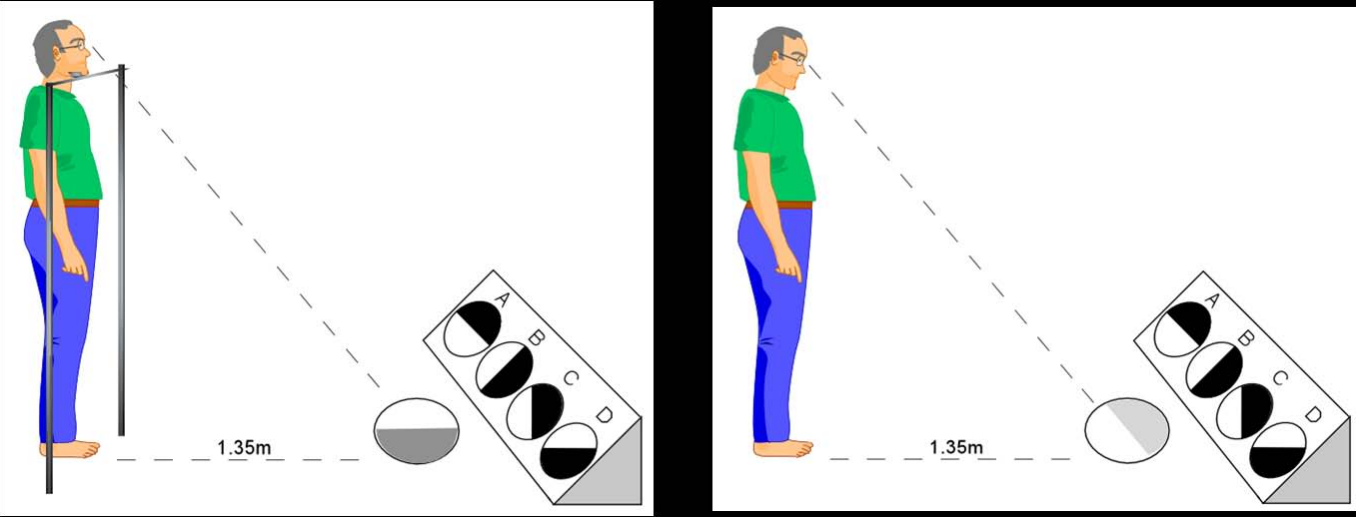
**The distance contrast sensitivity tests**. Panel A shows a participant (with his chin placed on a comfortable chinrest to prevent head movement) viewing the test plate through the lower (reading) segments of his glasses. Panel B shows the participant looking directly at the test stimulus through the upper (distance) segments of his glasses. Any difference in contrast vision between the two conditions is then shown to the participant as part of the counselling session.

At a second visit (approximately four weeks following the initial assessment) participants will be fitted with their glasses. An optometrist/counsellor will then demonstrate how multifocal glasses can impair the visual abilities required for detecting obstacles and judging depth. This will be achieved by administering the tests of distance edge contrast sensitivity and depth perception with participants viewing the visual stimuli through the upper and lower portion of their multifocal lenses and distance lenses [5]. The difference in upper and lower visual performance will be shown to the participant and the rationale for performing these tests will then be explained. Figure 2 shows the tests for measuring upper and lower lens contrast sensitivity used in the counselling (Figure 2).

**Figure 3:**
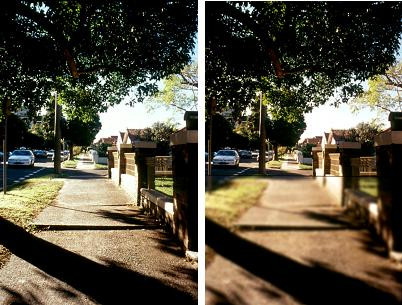
**Simulated view of street scene as viewed through single-lens distance (panel A) and bifocal glasses (panel B)**. The footpath misalignment (a commonly reported environmental factor involved in outdoor falls) is clearly seen in panel A, but blurred in panel B.

Participants will also be shown photographs of steps and streetscapes with and without simulated lower field blur to further reinforce why multifocal glasses may increase the risk of falls. Figure 3 shows the photographs of a simulated view of a street scene as viewed through single-lens distance (panel a) and bifocal glasses (panel b) (Figure 3). This counselling session will be conducted in line with the health belief model [20], and is considered crucial to convince older wearers of multifocal glasses that they are personally at risk of fall-related injuries and that the benefits of wearing single lens glasses outweighs the inconvenience of dealing with two pairs of glasses.

The optometrist/counsellor will then instruct participants to use their new single-lens glasses for most walking and standing activities, and in particular when undertaking the following activities: walking up or down stairs outside the home, walking in the street and at shopping centres, walking or standing in other people's homes and unfamiliar buildings, negotiating rough or uneven ground and getting off and on public transport. Multifocal glasses use will not be discouraged for seated tasks, especially those that require frequent changes in focal length such as driving, and indoor standing and walking tasks that require changes in focal depth where there is minimal risk (e.g. cooking and selecting items in the supermarket).

To assist with the swapping of glasses, participants will be provided with a "glasses cord" for wearing around the neck and/or a hard or cloth glasses holder for keeping the second pair of glasses in a pocket. Intervention group participants will also be provided with a booklet reinforcing the advice. Table 1 outlines the strategies that will used to maximise compliance throughout the trial (Table 1). Any perceived barriers to compliance will be recorded.

**Table 1:** Strategies to be used to maximise compliance

1	A strong evidence-based rationale for the intervention and clear recommendations will be provided.
2	The mechanism by which multifocal glasses can predispose to falls will be demonstrated during counselling using the example of the most commonly reported environmental factor involved in outdoor falls (Figure 2).

3	New glasses will be provided at no cost.

4	Perceived barriers to using two pairs of glasses will be identified and discussed during counselling.

5	An information brochure with recommendations and illustrations on appropriate use of glasses will be provided.

6	A hard/soft case glasses holder (to be worn around the neck and/or placed in the participant's pocket) to aid the swapping of glasses will be provided.

7	Second counselling sessions delivered over the phone or at home visits will address perceived barriers to use of glasses for participants with inadequate compliance, i.e. "never/occasionally" complying with the recommendations.

8	Written reminder cards will be provided with falls diaries and prompts will be made during follow-up telephone calls.

Participants in the control group will also have the same optometric examination as participants in the intervention group. For the estimated 80% of participants who will not need a change of prescription (n = 234), no new glasses will be prescribed. For the expected 20% who will require a prescription change (n = 56), updated multifocal lenses will be prescribed according to the above criteria and fitted at a second visit. Control group participants will be given no specific advice regarding the use of their glasses.

### Outcomes

#### Primary outcome measure

The primary outcome measure will be the number of participant falls in the 13-month follow-up period following randomisation (this allows for an average time of one month for the provision of single-lens glasses following randomisation and 12-month further follow-up). Falls will be defined as 'inadvertently coming to rest on the ground or other lower level with or without loss of consciousness, and other than as a consequence of sudden onset of paralysis, epileptic seizure, excess alcohol intake or overwhelming external force' [21]. All participants will receive 13 calendars and questionnaires at the time of the baseline assessment. Participants will be asked to record falls (as well as information regarding any fall injuries and need for medical care) on the calendars and return them in pre-paid envelopes to the research centre each month. Participants who do not return calendars will be telephoned to ask for the information. Staff who receive falls calendars, make follow-up phone calls and enter data will be unaware of group allocation.

#### Secondary outcome measures

Secondary outcome measures will include physical activity levels, falls efficacy and quality of life assessed at baseline and 12 months. These measures are included to determine whether, as a result of fewer falls, the intervention has beneficial effects on an older person's abilities and quality of life. *Physical activity levels *will be assessed with the Adelaide Activities Profile, a validated scale which measures lifestyle activities using 21 items which generate four domains (domestic chores, household maintenance, service to others, and social activities) [14]. Physical and emotional wellbeing will be assessed using the *SF 12 Version 2 *composite physical and mental scores [15]. Falls efficacy will be assessed using the *FES-I *[16] in which level of concern about falling when carrying out 16 activities is rated on a 4-point scale.

### Compliance and adverse events

*Compliance *in the intervention group will be measured monthly by asking how often the participants complied with recommendations about wearing single-lens distance glasses when undertaking activities such as walking outdoors and descending steps etc. Reporting of the type of glasses worn during any fall will be recorded on the same questionnaire. Participants will be asked to return their compliance questionnaires in a separate pre-paid envelope to the falls calendar to the research centre each month. Staff who receive compliance questionnaires, make follow-up phone calls and enter data will be unaware of the occurrence of any participant falls. Any perceived barriers to compliance will be recorded at baseline and throughout the trial.

*Adverse effects *Falls occurring as a result of switching from single-lens to multifocal glasses or vice versa while undertaking an activity or any non-fall adverse outcome occurring as a result of the intervention will be recorded on the falls calendars.

### Sample size calculation

Approximately 580 participants (290 per group) will be recruited. The study will have 80% power to detect as significant at the 5% level a 23% reduction in the rate of falling (i.e. an IRR of 0.77 using negative binomial regression analysis) in the 13-month follow-up period. This represents a reduction from 0.85 falls/person (an established rate of falling in a high-risk control group equivalent to our sample) [22,23] to 0.65 falls/person in the intervention group. This allows for an 8% loss to follow-up defined as withdrawing from the study and/or not completing the falls diaries, 70% compliance in the intervention participants and a rate of 0.57 falls/person among those complying with the intervention

### Statistical analysis

The primary analysis will be by intention to treat. The number of falls per person-year will be analysed using negative binomial regression to estimate the difference in falls rates between the two groups, adjusted for confounding variables if required. This analysis takes into account all falls during the trial, and the distribution of falls, which is Poisson-like but containing a wider, higher tail in the distribution [24]. Three *a priori *subgroup analyses will be performed using a test for statistical interaction to assess whether the intervention effect size differs according to: (i) baseline physiological falls risk [18]; (ii) number of participant-reported falls in the year prior to the trial; and (iii) participant-reported outdoor activity levels during the trial as assessed with the AAP [14]. Secondary analyses will assess the effects of the intervention on falls that occur (i) within and (ii) beyond the participants' homes. Longitudinal mixed models will be used to assess the effect of group allocation on the continuously-scored secondary outcome measures. Predictors of adherence will be established using multivariate modelling techniques. Analyses will be conducted using the SPSS [25] and Stata [26] computer programs.

## Discussion

Falls are the leading cause of injury-related death and hospitalisation in older persons [26]. Falls can also result in disability, restriction of activity and fear of falling – all of which reduce quality of life and independence [27,28]. Furthermore, falls can contribute to the placement of an older person into institutional care [28]. There is mounting evidence that multifocal glasses impair vision, hinder obstacle avoidance and increase the risk of falls in older people.

There are over 2.4 million people over 65 years of age in Australia, one-third of whom fall annually at least once [27]. Given that 52% of older people rely solely on multifocal glasses [1] and only 1% use separate reading and distance glasses [1], over one million Australians could potentially benefit from this trial immediately. Current guidelines recognise the importance of vision in falls and recommend that older people not walk while wearing multifocal glasses [29,30]. It is worth noting, however, that this recommendation also removes the correction of distance refractive error which may increase falls risk in some people. Our intervention, which involves providing multifocal glasses wearers with an additional pair of single-lens distance glasses for high-risk situations that increase postural threats (such as walking on stairs and in unfamiliar outdoor settings), will avoid this problem.

Thus, this program is optimally designed to address the major problem of falls in older people and could be readily translated into clinical practice. The study we are undertaking is designed and adequately powered to assess the effect of the program. There will be far-reaching benefits for older people, their carers and the community if this program can be demonstrated to assist in reducing the number of falls in this high-risk population.

## Conclusion

The study will determine the impact of providing single-lens glasses, with advice about appropriate use, on preventing falls in older regular wearers of multifocal glasses.

## Competing interests

The authors declare that they have no competing interests.

## Authors' contributions

This manuscript was drafted by SL and MH. The other authors are also actively involved in the study. Many contributed to the writing of the grant application for this project which was funded by the Australian National Health and Medical Research Council in 2005. The grant application formed the basis for this manuscript. All authors contributed to the manuscript's critical review and approved the final version.

## Pre-publication history

The pre-publication history for this paper can be accessed here:


